# Haploinsufficiency due to a novel *ACO2* deletion causes mitochondrial dysfunction in fibroblasts from a patient with dominant optic nerve atrophy

**DOI:** 10.1038/s41598-020-73557-4

**Published:** 2020-10-07

**Authors:** Marie Anne-Catherine Neumann, Dajana Grossmann, Simone Schimpf-Linzenbold, Dana Dayan, Katarina Stingl, Reut Ben-Menachem, Ophry Pines, François Massart, Sylvie Delcambre, Jenny Ghelfi, Jill Bohler, Tim Strom, Amit Kessel, Abdussalam Azem, Ludger Schöls, Anne Grünewald, Bernd Wissinger, Rejko Krüger

**Affiliations:** 1 Luxembourg Centre for Systems Biomedicine (LCSB), University of Luxembourg, Esch-sur-Alzette, Luxembourg; 2 Faculty of Medicine, RWTH Aachen University, Aachen, Germany; 3 CeGaT GmbH and Praxis für Humangenetik Tübingen, Tübingen, Germany; 4 Institute for Ophthalmic Research, Centre for Ophthalmology, University of Tuebingen, Tübingen, Germany; 5 School of Neurobiology, Biochemistry and Biophysics, George S Wise Faculty of Life Sciences, Tel Aviv University, Tel Aviv, Israel; 6 Centre for Ophthalmology, University Eye Hospital, University of Tübingen, Tübingen, Germany; 7 Department of Microbiology and Molecular Genetics, IMRIC, Faculty of Medicine, Hebrew University, Jerusalem, Israel; 8 NUS-HUJ-CREATE Program and the Department of Microbiology, School of Medicine, National University of Singapore, Singapore, Singapore; 9 Institute of Human Genetics, Helmholtz Zentrum Muenchen, Neuherberg, Germany; 10 Department of Neurology and Hertie-Institute for Clinical Brain Research, University of Tübingen, Tübingen, Germany; 11 German Center for Neurodegenerative Diseases (DZNE), Tübingen, Germany; 12 Institute of Neurogenetics, University of Lübeck, Lübeck, Germany; 13 Transversal Translational Medicine, Luxembourg Institute of Health (LIH), Strassen, Luxembourg; 14 Parkinson Research Clinic, Centre Hospitalier de Luxembourg (CHL), Luxembourg, Luxembourg

**Keywords:** Genetics, Neuroscience, Neurology

## Abstract

ACO2 is a mitochondrial protein, which is critically involved in the function of the tricarboxylic acid cycle (TCA), the maintenance of iron homeostasis, oxidative stress defense and the integrity of mitochondrial DNA (mtDNA). Mutations in the *ACO2* gene were identified in patients suffering from a broad range of symptoms, including optic nerve atrophy, cortical atrophy, cerebellar atrophy, hypotonia, seizures and intellectual disabilities. In the present study, we identified a heterozygous 51 bp deletion (c.1699_1749del51) in *ACO2* in a family with autosomal dominant inherited isolated optic atrophy. A complementation assay using *aco1*-deficient yeast revealed a growth defect for the mutant ACO2 variant substantiating a pathogenic effect of the deletion. We used patient-derived fibroblasts to characterize cellular phenotypes and found a decrease of ACO2 protein levels, while ACO2 enzyme activity was not affected compared to two age- and gender-matched control lines. Several parameters of mitochondrial function, including mitochondrial morphology, mitochondrial membrane potential or mitochondrial superoxide production, were not changed under baseline conditions. However, basal respiration, maximal respiration, and spare respiratory capacity were reduced in mutant cells. Furthermore, we observed a reduction of mtDNA copy number and reduced mtDNA transcription levels in ACO2-mutant fibroblasts. Inducing oxidative stress led to an increased susceptibility for cell death in ACO2-mutant fibroblasts compared to controls. Our study reveals that a monoallelic mutation in ACO2 is sufficient to promote mitochondrial dysfunction and increased vulnerability to oxidative stress as main drivers of cell death related to optic nerve atrophy.

## Introduction

Optic atrophy is found either as an isolated disease or as part of syndromic disorders^1,2^. Maternally inherited Leber’s hereditary optic neuropathy (LHON) and autosomal dominant atrophies are the most common inherited optic atrophies, accounting for 30–50% of inherited optic neuropathies^3,4^. These disorders can be caused by mutations either in the nuclear or in the mitochondrial genome, and most frequently involve genes linked to mitochondrial function^5–8^.

To date, a number of families or isolated patients with mutations in the *Aconitase 2* (*ACO2)* gene were described, clinically presenting with neurodegenerative or metabolic disease of variable severity. *ACO2*-linked pathologies are inherited as an autosomal recessive trait, as all described patients carry homozygous or compound heterozygous missense or frameshift mutations, respectively^5,9–14^.

*ACO2* is a nuclear gene localized on human chromosome 22q13.2, which encodes for the mitochondrial monomeric protein Aconitase 2 (ACO2). ACO2 catalyzes the conversion of citrate to isocitrate within the tricarboxylic acid cycle (TCA)^15^. This enzymatic reaction is catalyzed by an iron (Fe)–Sulphur (S) cluster, which contains one particularly labile iron ion and is characteristic for the aconitase superfamily^16,17^. Furthermore, the Fe–S cluster functions not only in providing the enzymatic activity of ACO2 in the TCA cycle, but also senses and regulates mitochondrial iron homeostasis^18^ via the enzymatic conversion of citrate to isocitrate. The down-regulation of ACO2 preserves a higher level of citrate, which transports iron into mitochondria and thereby ensures the required iron levels^19^. Iron is an essential element and crucial for many processes, but iron accumulation also promotes oxidative stress. As it is especially susceptible to different inducers of oxidative stress^20,21^, ACO2 functions as a sensitive redox sensor^22^. This sensitivity might explain the observed accumulation of ACO2 during oxidative stress, subsequently causing mitochondrial oxidative damage^23,24^.

Furthermore, ACO2 was described to play a crucial role for the maintenance of mtDNA in human cells^10,13^ and in yeast, independent of its enzymatic activity in the TCA^25^. Due to its crucial function in central metabolic pathways, ACO2 is reported to play a role in a various number of metabolic diseases such as diabetes^26^ or oncological ailments^27,28^, as well as neurodegenerative diseases^5,9–13^.

In the present study, we identified a heterozygous 51 bp in-frame deletion in *ACO2* in a family with dominant inherited isolated optic atrophy. We used fibroblasts derived from the index patient in order to characterize mitochondrial phenotypes. Fibroblasts expressing mutant ACO2 revealed decreased levels of ACO2 protein, a reduction of mtDNA copy number and decreased mitochondrial respiration. We also observed a higher vulnerability to oxidative stress in these cells, leading to increased cell death. From these results, we conclude that impaired mitochondrial function caused by heterozygous mutations in the ACO2 gene are sufficient to cause neurodegeneration leading to optic nerve atrophy observed in the patient.

## Results

### Identification of the deletion c.1699_1749del51 in the ACO2 gene in a patient with dominant inherited optic nerve atrophy

**Figure 1:**
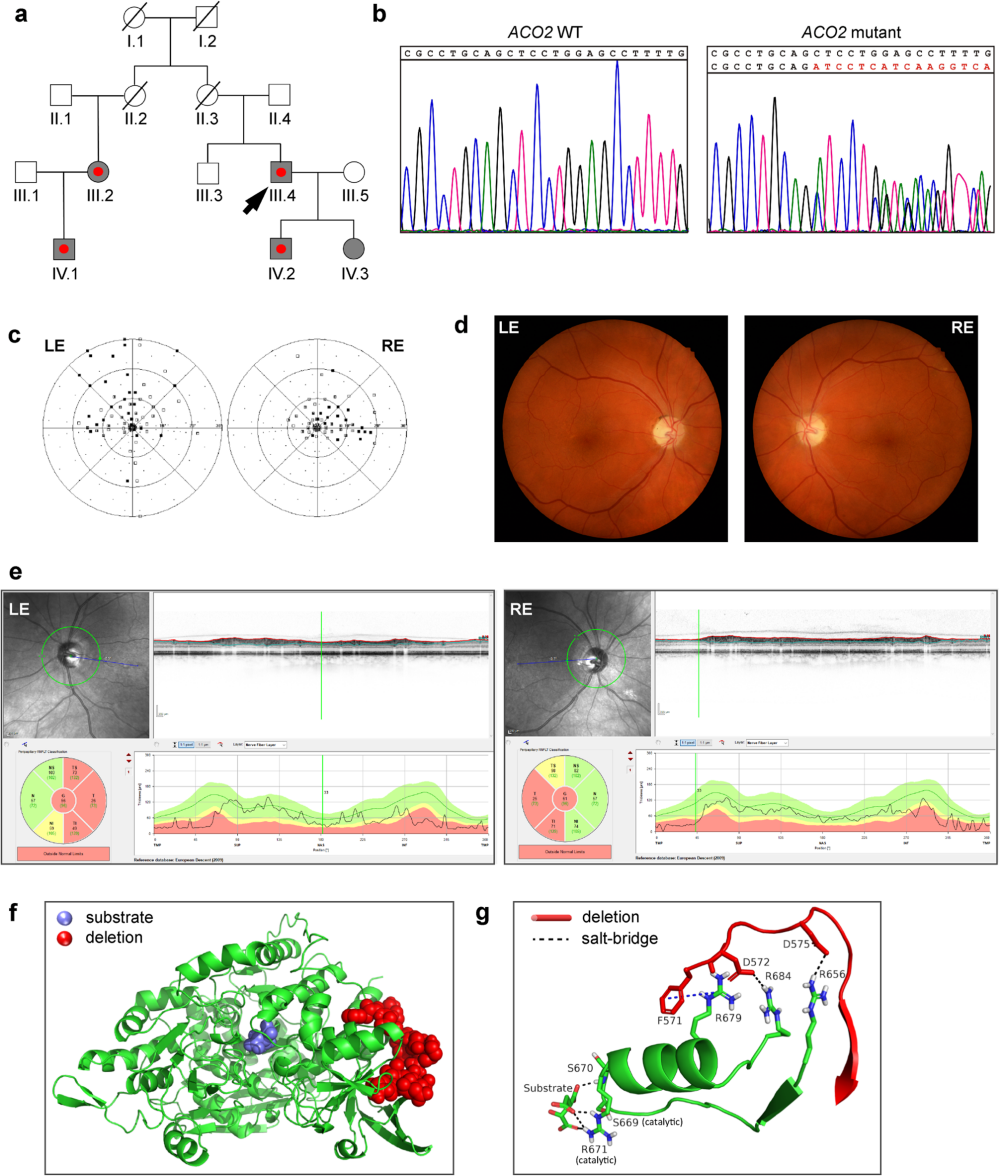
Identification of the deletion c.1699_1749del51 in the *ACO2* gene in a patient with dominant inherited optic nerve atrophy. (**a**) Family pedigree of the patient. Grey symbol: family members carrying the *ACO2* deletion c.1699-1749del51. Red point: clinically affected family member with optic atrophy. Arrow head points to the index patient, from whom we obtained skin fibroblasts for the present study. Deceased individuals are marked with a line. (**b**) Results of Sanger sequencing of genomic DNA obtained from a healthy individual (*ACO2* WT) and the index patient (*ACO2* mutant), confirming the identified deletion c.1699-1749del51. (**c**) Visual field from the ophthalmologic examination of the patient (done in 2011) showing central scotoma of the left (LE) and the right eyes (RE). Dots represent defects surrounded by normal visual field, called scotomas (range: white > 5 dioptries (dB) to black > 30 dB). (**d**) Funduscopy of the ophthalmologic examination (done in 2011). Temporal paleness of the optic nerve in both eyes. (**e**) Retinal fibre layer of the optic nerve heads show thinning of the optical nerve fibers in the temporal regions via spectral domain optical coherence tomography (SD-OCT). The retinal fiber layer thickness is shown on the upper right side along the circular scan line (highlighted by green color on the upper left panel). The normal layer thickness is indicated by the green area on the lower right panel. The pathologically thinning of the layer in the temporal sectors of both eyes is indicated by yellow and red color. *N* nasal, *NS/NI* nasal superior/inferior, *T* temporal, *TS/TI* temporal superior/inferior. (**f**) Overall structure of the human ACO2 protein. The substrate-binding region is highlighted in blue. The region affected by the deletion is highlighted in red. (**g**) The stabilizing interactions between the segment 571–583 affected by the deletion (red backbone) and segment 656–684 (green), which passes through the active site and interacts with the substrate. Salt bridges are shown as black dashed lines, and the combined π-π and cation-π interactions are shown as blue dashed line.

We screened 9 unrelated German patients suffering from autosomal dominant optic nerve atrophy for mutations using whole exome sequencing (WES). For the selection of putative disease-causing genes, we considered a sub panel of genes associated with optic atrophy, including *OPA1*, *OPA3*, *TMEM126A*, *WFS1*, *MFN2*, *SPG7*, *ACO2*, *RTN4IP1* and *AFG3L2*, and identified one male individual with the heterozygous deletion c.1699_1749del51 in the *ACO2* gene (NCBI reference NM_001098.3; hereafter referred to as ACO2-mutant or ACO2.mut) (Fig. 1a; individual III.4). The deletion was subsequently validated in genomic DNA of the index patient by Sanger sequencing (Fig. 1b). The same deletion in *ACO2* was found in all three other clinically affected family members, including the index patient’s son (individual IV.2), his first-degree female cousin (individual III.2) and her son (individual IV.1). The index patient’s daughter (individual IV.3) also carried the mutation but did not show clinical signs of disease when examined at the age of 16 years (Fig. 1a). From the WES data, we could exclude mutations in *OPA1*, *OPA3*, *TMEM126A*, *WFS1*, *MFN2*, *SPG7*, *RTN4IP1* and *AFG3L2* and the common mtDNA mutations associated with LHON at positions 11778/*ND4*, 3460/*ND1* and 14484/*ND6* in the index patient.

The patient was 26 years old at diagnosis. Clinical examination of the patient was performed at the age of 55 years at the Institute for Ophthalmic Research and the Section for Clinical Neurogenetics of the University Clinic of Tübingen, Germany. The optic nerve atrophy manifested with a decreased visual acuity on the right eye (0.7 decimal, corresponding to approx. LogMAR 0.2), while the left eye was amblyopic in the course of esotropia with an acuity of 0.08 (decimal, LogMAR 2.1). During 30 years of disease progression, the best corrected visual acuity decreased to 0.2 (decimal, LogMAR 0.7) on the right eye, while remaining constant on the left eye. Visual field examination revealed central scotomas on both eyes (Fig. 1c).

Ophthalmological examination showed a distinct temporal pallor of the optic nerve on funduscopy, indicating an atrophy of the optic nerve, with otherwise normal morphological findings of the anterior and posterior eye segment (Fig. 1d). Examination of the retinal nerve fiber layer via spectral domain optical coherence tomography (SD-OCT) shows thinning of the optical nerve fibers in the temporal regions (Fig. 1e).

The outer retinal function for the central visual field assessed by multifocal electroretinogram, as well as by the full-field electroretinogramm, was normal. The outer retina of the macular area on OCT was normal.

Neurological examination revealed normal findings apart from visual deficits described above. Especially, there were no signs of cerebellar ataxia or cognitive deficits reported before in carriers of biallelic *ACO2* mutations^5,9–11,13,14^. Laboratory tests found increased levels of ferritin (79 µg/dl; norm: 3–30) with normal levels of iron and transferrin.

Quantitative cDNA analysis with RNA from whole blood of the index patient revealed no evidence of impaired transcript splicing (data not shown). Translation of mutant transcripts thus results in an in-frame deletion of 17 amino acids (p.567_583del17) in the ACO2 protein (XP_024308018.1) in a region which is conserved in eukaryotic mitochondrial aconitases.

Visualization of the overall structure of ACO2 shows that the deleted 571–583 segment lies far from the active site, and faces the surrounding solvent on one side (Fig. 1f). Still, the deletion is likely to affect the enzymatic activity of the protein because the deleted region includes several charged amino acids (FDKWDGKDLEDLQ), some of which form salt-bridges with key positions in the protein (Fig. 1g, black dashed lines).

This includes mainly the following: Two salt bridges between Asp-572 in segment 571–583 and two arginine residues (Arg-679 and Arg-684), and one salt bridge between Asp-575 in segment 571–583 and Arg-656. In addition, the evolutionary conserved Phe-571 interacts with the highly conserved Arg-679 via π–π and cation–π interactions (Fig. 1g, blue dashed lines).

The three arginine residues interacting with segment 571–583 are part of a long segment (656–684) that passes through the active site, and which interacts directly with the substrate. These interactions are mediated via Ser-670 and Arg-671.

The segment containing the two residues also contains Ser-669, which together with Arg-671 participate in catalysis. Deletion of segment 571–573 is likely to cause a movement of segment 656–684, and in turn disrupt its catalytically important interactions with the substrate. This may happen in at least two ways: (i) by removing the salt bridges and π interactions described above, resulting in segment 669–684 being free to move, or (ii) by removing the stabilizing electrostatic masking of the arginine residues by Phe-571 and Asp-572 + 575, which will ensue a strong repulsion between the arginine residues.

The repulsion between Arg-679 and Arg-684, both located on the same α-helix, is likely to distort the helix. In addition, the repulsion between Arg-679 + 684 and Arg-656, which is on a separate β-strand, is likely to push the first two away from the third, and in turn induce a movement of the entire segment (656–684). Since the catalytic residues located on this segment are accurately positioned to interact with the substrate and perform catalysis, the above distortion and movement of segment 656–684 are likely to disrupt substrate binding and catalysis.

### The deletion c.1699_1749del51 in the ACO2 gene leads to a growth defect of a Δaco1 yeast strain

**Figure 2:**
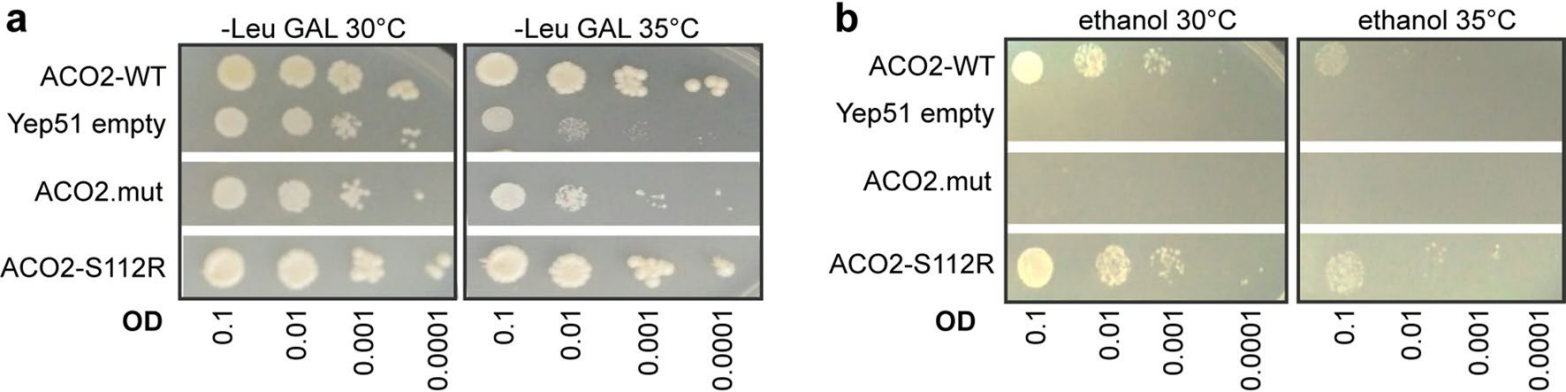
The deletion c.1699_1749del51 in the ACO2 gene leads to a growth defect of a Δaco1 yeast strain. (**a**) Drop dilution assay of yeast grown on galactose medium or (**b**) on ethanol medium at 30 °C or 35 °C and at different dilutions (optic densities (OD) ranging from 0.1, 0.01, 0.001 to 0.0001). Yeast cells with deletion of the *aco1* gene expressed either human ACO2 wild-type (WT) or the ACO2 deletion-mutant (ACO2.mut) or ACO2-S112R, respectively. Expression of an empty Yep51 vector served as negative control.

Aconitase is highly conserved across species and sequence alignment of human ACO2 (NP_001089.1)^29^ and *S. cerevisiae* ACO1 (NP_013407.1) generated by MUSCLE^30^ reveals a high similarity of both amino acid sequences (see Supplementary Fig. S1) of 66.41% (identity matrix created by Clusta12.1). We investigated the pathogenic potential of the *ACO2* mutations by complementation analysis in *S. cerevisiae* with deletion of the yeast *aco1* gene (*Δaco1*). Yeast cells were grown on galactose (Fig. 2a) or on ethanol medium (Fig. 2b) at different dilutions. When grown on galactose medium, yeast cells are forced to use mitochondrial respiration to maintain their energy supply^31,32^. Yeast devoid of *aco1*, expressing only the empty Yep51 vector showed a slight growth defect at 30 °C, while growth at 35 °C was severely affected. Expression of the human wildtype ACO2 or ACO2-S112R^9^ missense mutant rescued the growth defect of *Δaco1*. In contrast, expression of the p.567_583del17 mutant ACO2 (ACO2.mut) was not sufficient to rescue the growth defect of *Δaco1*, indicating that mitochondrial respiration was impaired by this mutation (Fig. 2a).

The growth of yeast cells on a two-carbon substrate like ethanol requires a functional TCA^32^. *Δaco1* yeast and *Δaco1* yeast expressing the p.567_583del17 mutant ACO2 were not able to grow on ethanol substrate, while *Δaco1* yeast expressing ACO2-WT or ACO2-S112R^9^ showed similar growth on ethanol substrate at 30 °C and 35 °C (Fig. 2b).

Overall, these results suggest that loss of *aco1* and the p.567_583del17 mutant ACO2 cause defects of the TCA and the mitochondrial respiration.

### Disease-associated ACO2 mutations cause impaired mtDNA maintenance

**Figure 3:**
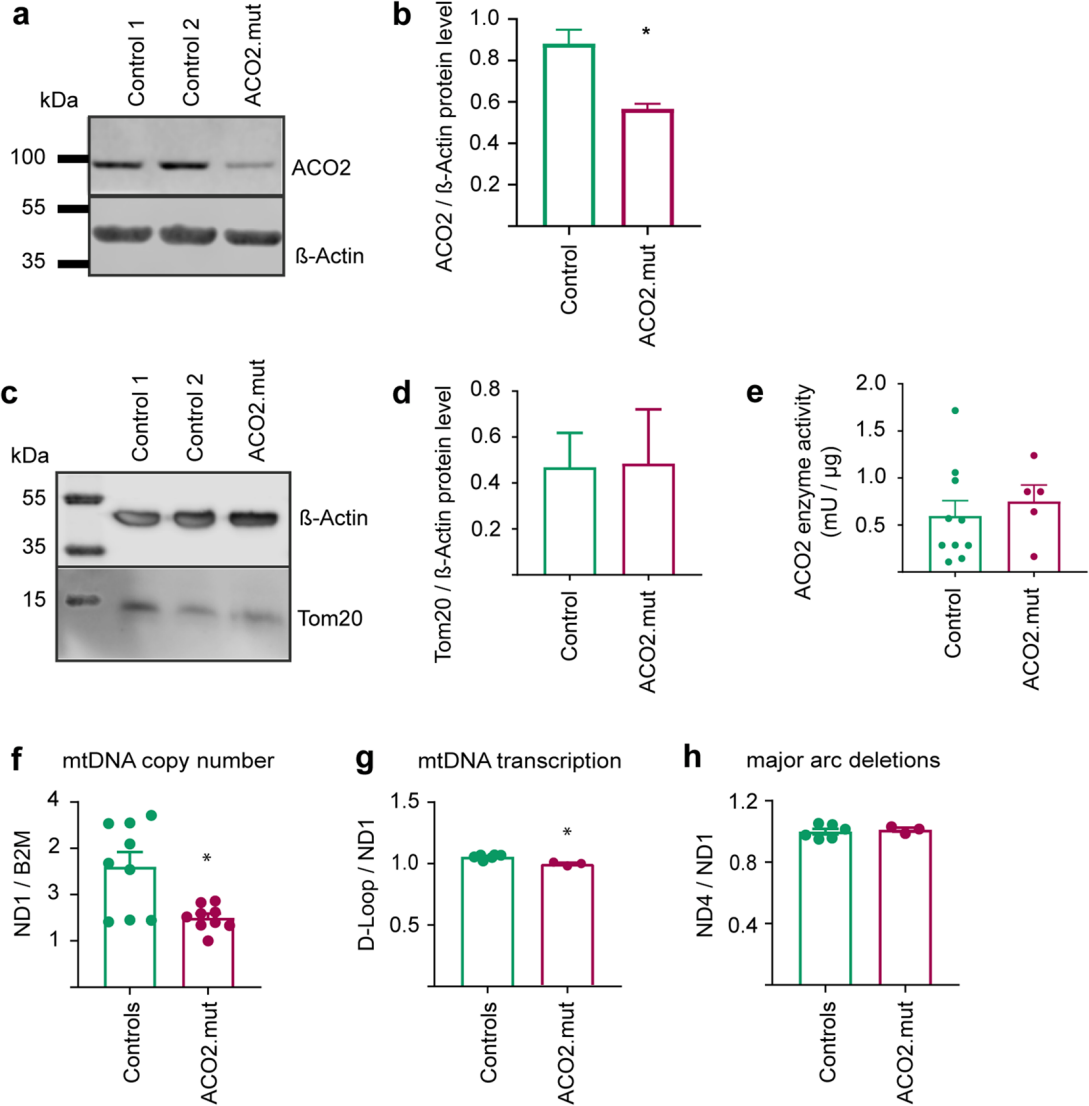
Disease-associated ACO2 mutations cause impaired mtDNA maintenance. (**a**) Representative image of Western blot analysis of ACO2 protein. (**b**) Quantification of Western blot analysis of ACO2 protein levels normalized to β-Actin. Significance calculated by Mann–Whitney test (n = 3). (**c**) Western blot image and (**d**) the corresponding quantification of Tom20 protein, normalized to β-Actin. Significance calculated by Mann–Whitney test (n = 3). (**e**) Biochemical measurement of ACO2 enzyme activity in mitochondrial fractions from fibroblasts. Significance calculated by Mann–Whitney test (n = 5). (**f**) mtDNA copy number was analyzed by RT-PCR and indicated as ratio of the copy numbers of the mitochondrial gene ND1 to the nuclear encoded gene B2M. Significance was calculated by Mann–Whitney test (n = 9). (**g**) mtDNA transcription was analyzed by RT-PCR and indicated as ratio of the copy numbers of the D-Loop to the mitochondrial gene ND1. Significance was calculated by Mann–Whitney test (n = 3). (**h**) Major arc deletions in the mitochondrial genome were analyzed by RT-PCR and indicated as the ratio of the copy numbers of ND4, which is located on the minor arc, to ND1, which is located on the major arc of the mtDNA. Significance was calculated by Mann–Whitney test (n = 3). All data were indicated as mean ± SEM. *p ≤ 0.05, **p ≤ 0.01, ***p ≤ 0.001.

We obtained fibroblasts from a skin biopsy of the patient and of healthy, age- and gender-matched control individuals. Western blot analyses revealed a significant decrease of ACO2 protein levels in ACO2-mutant fibroblast, compared to control fibroblasts (Fig. 3a,b).

As ACO2 is a mitochondrial protein, we also assessed the abundance of the mitochondrial marker protein Tom20 by Western blot (Fig. 3c). Here, we did not observe changes of protein levels between the fibroblast lines (Fig. 3d).

Given the reduced ACO2 protein levels in ACO2-mutant fibroblasts, we further determined ACO2 enzyme activity using a spectrophotometric assay. In order to avoid distortion of the assay by also measuring the enzyme activity of the cytosolic isoform Aconitase 1 (ACO1), we used mitochondrial fractions. Our results revealed that despite reduced protein levels, ACO2-mutant fibroblasts showed no alteration of overall ACO2 enzyme activity, using NADP+ as a substrate (Fig. 3e).

Together, our results suggest that the heterozygous 17 amino acid deletion in ACO2 likely leads to a reduction of ACO2 protein level, probably by increased degradation of the mutant protein. But apparently, the wild type proportion of ACO2 protein is sufficient to maintain the overall enzyme activity in patient-derived fibroblasts under baseline conditions.

Compound heterozygous mutations in *ACO2* were previously shown to cause a ~ 50% reduction of mtDNA copy number^10^, leading us to further investigate mtDNA maintenance in ACO2-mutant fibroblasts by RT-PCR. Our analysis revealed that mtDNA copy number (Fig. 3f) and mtDNA transcription (Fig. 3g) were significantly reduced in ACO2-mutant fibroblasts compared to control cells, while we did not observe changes of the amount of deletions in the major arc of the mitochondrial genome (Fig. 3h).

### ACO2-mutant fibroblasts display reduced mitochondrial respiratory function

**Figure 4:**
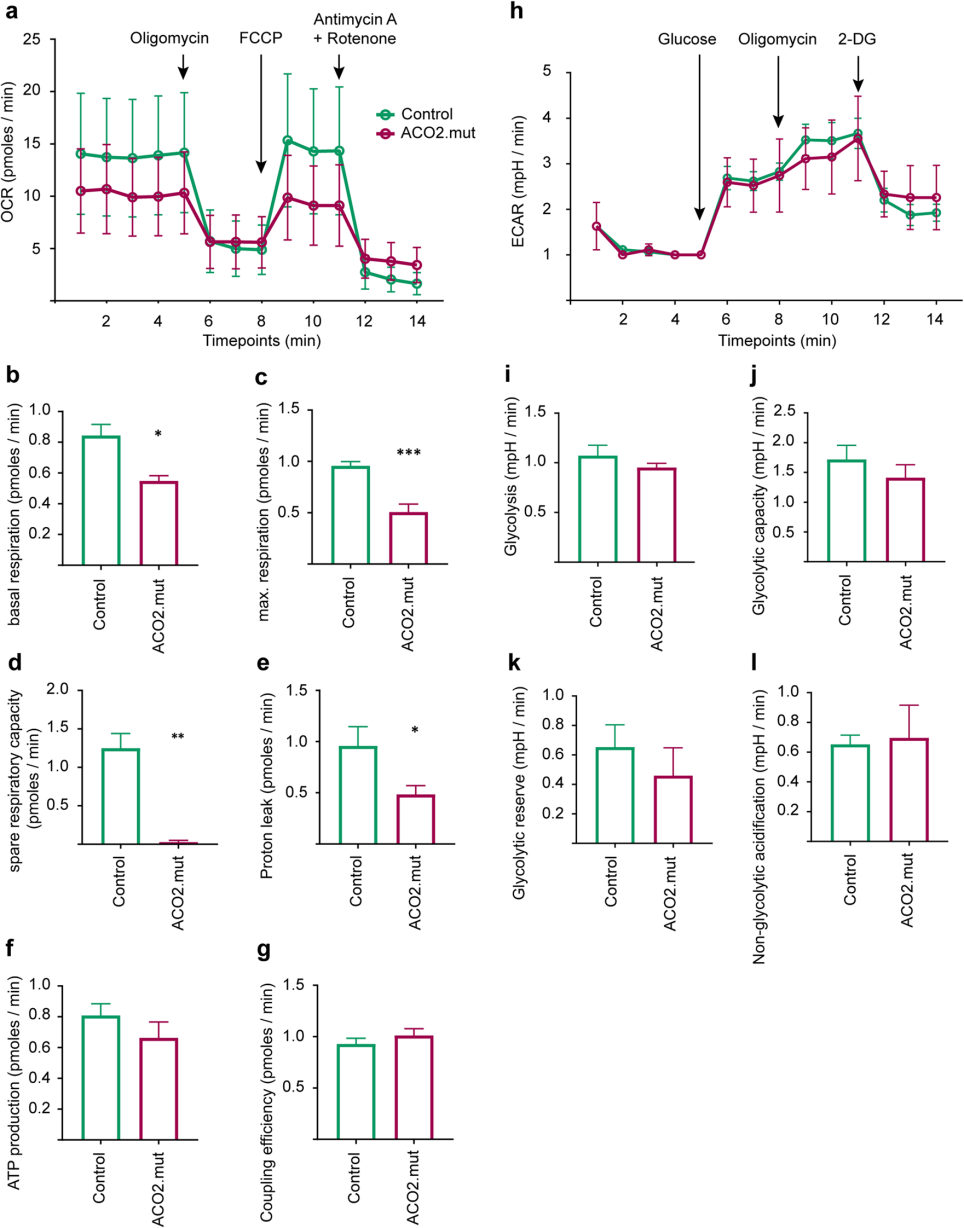
ACO2-mutant fibroblasts display reduced mitochondrial respiratory function. (**a**) Overview of measurement of oxygen consumption rate (OCR) in whole fibroblasts sequentially treated with 1 µM Oligomycin, 250 nM FCCP and 5 µM Antimycin A + Rotenone. OCR data were normalized to the total protein concentration in each well after cell lysis. Data indicated as mean ± SEM, (n = 5). (**b**) Basal respiration, (**c**) maximal respiration, (**d**) spare respiratory capacity, (**e**) proton leak, (**f**) ATP production and (**g**) coupling efficiency calculated from OCR data shown in (**a**). Data indicated as mean ± SEM. Significance calculated by Mann–Whitney test, *p ≤ 0.05, **p ≤ 0.01, ***p ≤ 0.001, (n = 5). (**h**) Overview of measurement of the extra-cellular acidification rate (ECAR) in whole fibroblasts. During measurement, cells were sequentially treated with 1 mM Glucose, 10 µM Oligomycin and 10 mM 2-deoxyglucose (2-DG). ECAR data were normalized to the total protein concentration in each well after cell lysis. Data indicated as mean ± SEM, (n = 3). (**i**) Glycolysis rate, (**j**) Glycolytic capacity, (**k**) Glycolytic reserve and (**l**) non-glycolytic acidification were calculated from ECAR data shown in (**h**). Data indicated as mean ± SEM. * p ≤ 0.05, **p ≤ 0.01, ***p ≤ 0.001, Mann–Whitney test (n = 3).

ACO2 is an important enzyme of the TCA and is therefore critically involved in mitochondrial metabolism, which was assessed by measurement of the oxygen consumption rate (OCR) in whole fibroblasts (Fig. 4a). The OCR measurement enables the calculation of several parameters of the respiratory chain function: Basal respiration (Fig. 4b), maximal respiration (Fig. 4c), spare respiratory capacity (Fig. 4d) and proton leak (Fig. 4e) were significantly reduced in ACO2-mutant fibroblasts, compared to control fibroblasts, whereas ATP production (Fig. 4f) and the coupling efficiency (Fig. 4g) were not changed. These results suggest that mitochondrial respiration is impaired in fibroblasts under baseline conditions.

The assessment of mitochondrial respiration was further completed by measurements of the extracellular acidification rate (ECAR; Fig. 4h), which allows conclusions on glycolysis. Results show that neither glycolysis (Fig. 4i), glycolytic capacity (Fig. 4j), glycolytic reserve (Fig. 4k), nor non-glycolytic acidification (Fig. 4l) were changed in ACO2-mutant fibroblasts.

From these results, we conclude that ACO2-mutant fibroblasts are able to maintain their energy supply by glycolysis under normal cell culture conditions. By contrast, the observed reduction of mitochondrial respiration in the presence of mutant ACO2 suggests that patient cells may not be able to adapt their metabolism to challenging conditions.

### ACO2-mutant fibroblasts are more susceptible to oxidative stress

**Figure 5:**
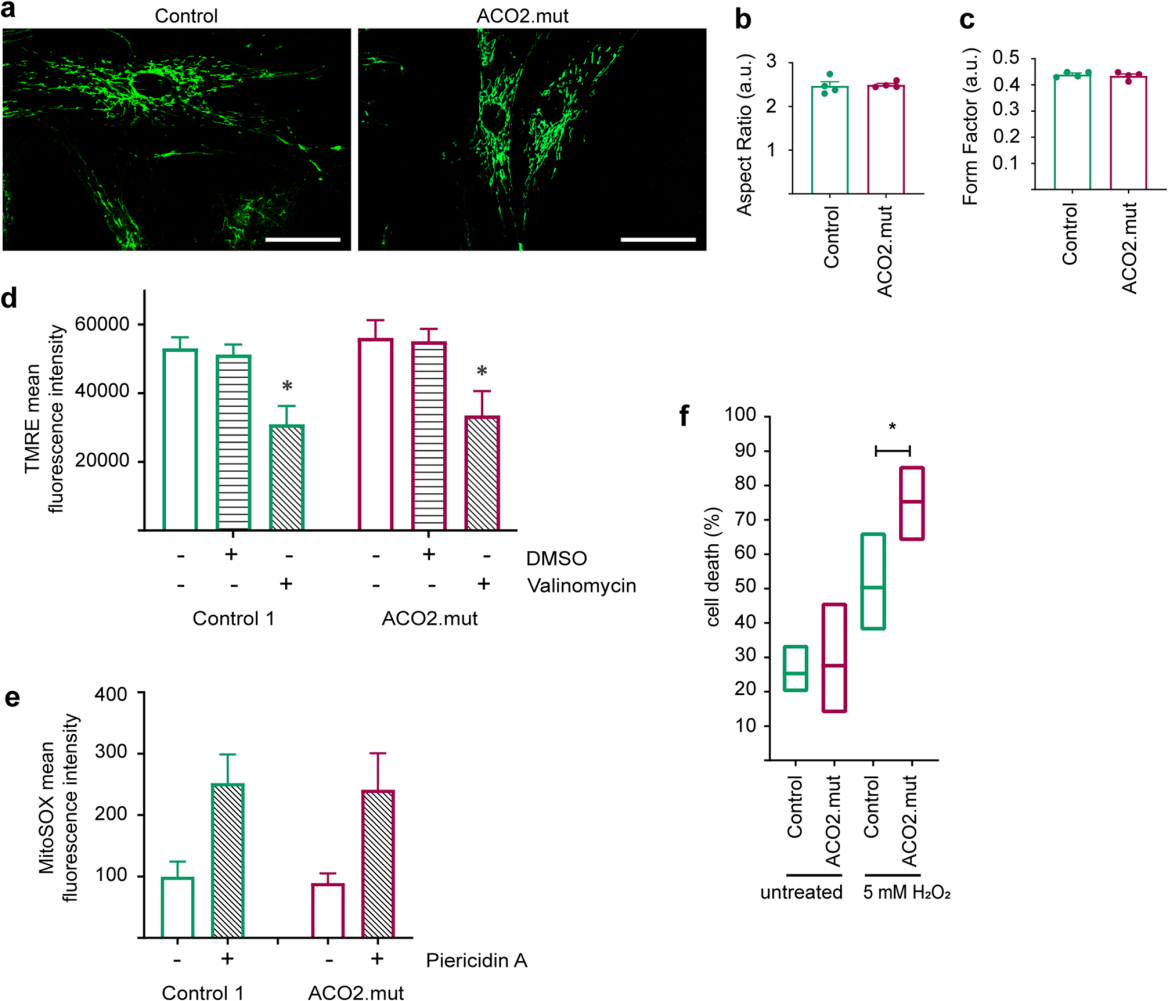
ACO2-mutant fibroblasts are more susceptible to oxidative stress. (**a**) Representative images of control and ACO2-mutant fibroblasts stained with MitoTracker green FM for live cell imaging of mitochondria. Images were obtained with a 40 × objective. Scale bars indicate 50 µm. Images were used to analyse parameters of mitochondrial morphology, indicating (**b**) mitochondrial length, reflected by aspect ratio and (**c**) mitochondrial branching, reflected by form factor. Data indicated as mean ± SEM (n = 4). (**d**) Mitochondrial membrane potential of fibroblasts was analyzed by FACS. Cells were treated with 5 nM Valinomycin for 14 h in order to decrease the mitochondrial membrane potential and afterwards stained with TMRE. Data indicated as mean ± SEM, significance calculated by 2way ANOVA with post hoc Tukey’s multiple comparison test (n = 4). (**e**) Mitochondrial superoxide production was measured in fibroblasts using FACS analysis of MitoSOX staining. Cells were first treated with 20 nM Piericidin A for 14 h in order to inhibit the activity of the respiratory chain complex I. Data indicated as mean ± SEM, significance calculated by Mann–Whitney test (n = 3). (**f**) Cell viability was assessed using the Lactic Acid Dehydrogenase (LDH) assay. Fibroblasts were incubated with 5 mM H_2_O_2_ for 4 h and afterwards the proportion of cell death was calculated from the amount of released LDH (n = 5). Data indicated as mean ± min./max. values. Significance calculated by Mann–Whitney test. *p ≤ 0.05, **p ≤ 0.01, ***p ≤ 0.001.

The observed alterations of mitochondrial respiration in ACO2-mutant fibroblasts led us to investigate the consequence of mutant ACO2 on mitochondrial integrity. First, we analyzed mitochondrial morphology in fibroblasts under baseline conditions (Fig. 5a) and found no changes of mitochondrial aspect ratio, indicative for length (Fig. 5b), or mitochondrial form factor, indicating branching (Fig. 5c), in ACO2-mutant fibroblasts compared to control cells.

Also, we did not observe alterations of mitochondrial membrane potential in ACO2-mutant fibroblasts using TMRE staining, neither under baseline conditions, nor under Valinomycin treatment (Fig. 5d). Furthermore, we did not observe changes of mitochondrial superoxide production in ACO2-mutant fibroblasts, compared to control cells, when using MitoSOX staining under baseline conditions and under complex I inhibition with Piericidin A (Fig. 5e).

Finally, we investigated the effect of oxidative stress on cell viability by using the LDH assay. Cell viability in ACO2-mutant fibroblasts was comparable to control fibroblasts under baseline conditions, however, after oxidative challenge by treatment with H_2_O_2_, we observed a significant increase of cell death in ACO2-mutant fibroblasts compared to control cells (Fig. 5f).

Together, the results suggest that mutations in ACO2 lead to an increased susceptibility to oxidative stress.

### ACO2 enzyme activity correlates with the severity of the clinical phenotype, but not with the protein amount of ACO2

**Figure 6:**
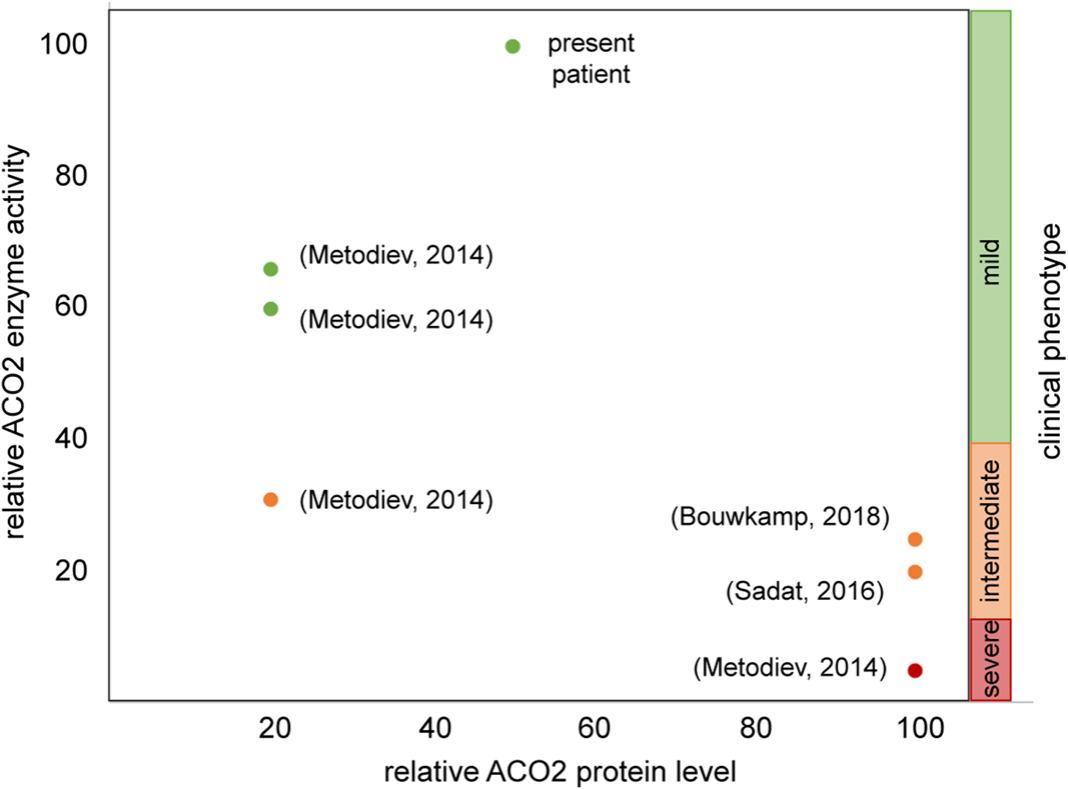
ACO2 enzyme activity correlates with the severity of the clinical phenotype, but not with the protein amount of ACO2. Overview of patients with mutations in *ACO2* from different studies, showing the correlation between ACO2 enzyme activity, protein level and severity of the clinical phenotype. Patients with mild clinical phenotype are represented with green dots, patients with intermediate phenotype are highlighted by orange dots and patients depicted by red dots show severe clinical phenotypes.

A number of studies reported cases of patients carrying mutations in the *ACO2* gene, including analyses in patient-derived fibroblasts. From the available literature, we noticed that the enzymatic activity of mutant ACO2 does not correlate with the protein level, fitting to our observation of reduced ACO2 protein level (Fig. 3a,b) in spite of unchanged enzyme activity (Fig. 3e). Metodiev and colleagues described clinical presentation and cellular phenotypes in families with different mutations in *ACO2* and found that the compound heterozygous mutations c.220 C>G (p.Leu74Val)/c.1981 G>A (p.Gly661Arg) results in 20% protein level and 66% enzyme activity, while the homozygous mutation c.776 G>A (p.Gly259Asp) does not affect protein levels, but reduced enzyme activity to 5%^5^. Protein levels were reduced to 20% and enzyme activity to 31% in a compound heterozygous carrier of the mutations c.2208 G>C (p.Lys736Asp)^5^. Other studies also reported unchanged ACO2 protein levels, while the enzyme activity was reduced to 20% (compound heterozygous mutations c.2135 C>T; p.Pro712Leu^10^), or 25% (homozygous c.1240T>G; p.Phe414Val^33^), respectively (Fig. 6).

We blotted the ACO2 enzyme activity against the protein levels reported in several studies and noticed that the level of ACO2 enzyme activity seems to correlate with the severity of the clinical phenotype (Fig. 6). Patients with higher enzymatic activity display milder clinical symptoms, e.g. a patient carrying compound heterozygous mutations c.220 C>G (p.Leu74Val)/c.1981 G>A (p.Gly661Arg) showed 66% enzyme activity in fibroblasts and displayed optic atrophy^5^, while the homozygous mutation c.776 G>A (p.Gly259Asp) was associated with 5% enzyme activity and severe symptoms including optic atrophy, ataxia, cerebellar atrophy and seizures^5^. Fitting to this observation, the patient described in the present study presented with an isolated optic atrophy (Fig. 1) and an unaffected ACO2 enzyme activity in patient-derived fibroblasts (Figs. 3e, 6).

## Discussion

In the present study, we provide first evidence for haploinsufficiency of the *ACO2* gene as a cause of autosomal dominant isolated optic atrophy. We used WES to identify potential disease-causing mutations and found a novel heterozygous deletion, c.1699-1749del51, in the *ACO2* gene, resulting in a shortened ACO2 protein with an internal 17 amino acid deletion.

Mutations in *ACO2* have been described in the context of early fatal or neurodegenerative disease in homozygous or compound heterozygous state. The clinical symptoms range from mildly affected patients, suffering from isolated optic atrophy^9^, to severely diseased patients, presenting with cortical or cerebellar atrophy, hypotonia, seizures or intellectual disabilities starting in early infancy^5,9–13^. Based on the genetic results, we reason that the identified heterozygous c.1699_1749del51 *ACO2* mutation causes an autosomal dominant inherited isolated optic atrophy with reduced penetrance in the herein described family.

The pathogenic nature of the *ACO2* deletion c.1699_1749del51 identified in our study was further substantiated by performing a yeast complementation assay. The deletion of the mitochondrial *aco1* gene caused a severe growth defect on galactose medium as well as on ethanol medium. Complementation with a vector expressing the ACO2-mutant variant (*ACO2* c.1699_1749del51) was not sufficient to rescue this phenotype, indicating that both, mitochondrial respiration and the TCA cycle were affected. Similar phenotypes of *aco1*-deficient yeast complemented with different *ACO2* variants were reported in previous studies^5,9,25^.

The biochemical analysis of mitochondrial function in patient-derived fibroblasts and Δ*aco1*-yeast highlights the pathogenic relevance of the herein described *ACO2* c.1699-1749del51 deletion. It is worth noting that expression of the S112R mutation showed at least a partial complementation of Δ*aco1*-yeast, notwithstanding that this mutation was previously described to cause a recessively inherited syndrome of infantile cerebellar-retinal degeneration^9^.

Mitochondrial aconitase is an evolutionary strongly conserved protein that is involved in the TCA cycle and in the maintenance of mitochondrial function. Therefore, we used patient-derived fibroblasts to further analyze the cellular mechanisms of the underlying pathology.

Recent studies suggested that the ACO2 enzyme activity in patient-derived cells seem to generally correlate with the severity of clinical symptoms^5,11,13^. Indeed, the ACO2 enzyme activity varied considerably in the different studies, ranging from 5 to > 60% in cells derived from patients with either homozygous or compound heterozygous mutations, and patients with higher ACO2 enzyme activity presented with milder clinical phenotypes^5,9,10,13^. This finding fits to the comparatively mild clinical phenotype of isolated optic nerve atrophy in the herein described patient and the unaffected ACO2 enzyme activity in the patient-derived fibroblasts.

In contrast, several studies showed that ACO2 enzyme activity does not correlate with ACO2 protein levels. In 2014, Metodiev and colleagues analyzed ACO2 protein levels and enzyme activity in fibroblasts from patients with ACO2 mutations. Cells with the lowest levels of ACO2 protein (~ 20%) showed the highest levels of remaining mutant ACO2 enzyme activity (~ 30% to > 60%, respectively), while fibroblasts expressing the highest levels of ACO2 protein (unchanged levels compared to control fibroblasts) had the lowest enzyme activities (~ 5%)^5^. Additionally, the studies of Sadat et al*.* and Bouwkamp et al*.* showed that ACO2 enzyme activity was reduced to 20% or 25%, respectively, while the ACO2 protein levels were unchanged in patient-derived fibroblasts^10,33^.

Despite the fact that ACO2 enzyme activity was not significantly affected in fibroblasts derived from the patient, the mitochondrial respiration was impaired. Basal respiration, maximal respiration and spare respiratory capacity were significantly decreased, suggesting that ACO2-mutant cells have a reduced capacity to cope with metabolically demanding conditions. Sadat and colleagues previously observed a similar impairment of mitochondrial respiration in ACO2-mutant patient-derived fibroblasts, accompanied with a 50% reduction of mtDNA copy number^10^.

Hence, it was not surprising to find that fibroblasts with mutations in ACO2 in this study showed a 50% reduction of mtDNA copy number, which was likely the cause of impaired mitochondrial respiration. In yeast, the mitochondrial ACO1 protein, which corresponds to human ACO2, was found to be crucial for mtDNA maintenance. A diploid yeast strain with heterozygous depletion of *aco1* showed drastic reduction of mtDNA abundance^25^. This function of ACO1 was independent of the ACO1 catalytic activity and rather depending on retrograde metabolic pathways^25^. Therefore, we conclude that optic nerve atrophy may be caused by heterozygous mutations in the *ACO2* gene independent of its enzymatic activity. We postulate that the reduction of ACO2 protein levels leads to defects in mtDNA maintenance, which subsequently impair mitochondrial respiratory function.

In addition, we further investigated mitochondria-related phenotypes by assessment of the ECAR, which indicates glycolytic function, mitochondrial morphology, mitochondrial membrane potential and mitochondrial superoxide production, none of which showed changes in ACO2-mutant fibroblasts compared to control fibroblast lines. These results are not surprising given the fact that fibroblasts are able to maintain their energy demand by glycolysis.

We finally assessed cell viability in our fibroblast lines and were not able to detect differences at baseline conditions. However, when cells were challenged with oxidative stress using H_2_O_2_ treatment, ACO2-mutant fibroblasts showed a markedly increased level of cell death compared to control cells. ACO2 was previously described to be sensitive to oxidative stress^20,21^ and to play a role in oxidative stress-related pathways^22–24^. In 2011, Cantu et al. showed that paraquat-induced inhibition of ACO2 caused an increased production of H_2_O_2_ and ferrous iron accumulation with subsequent increased apoptosis of rat dopaminergic N27 cells. The authors concluded that ACO2 protects cells from apoptosis by the regulation of H_2_O_2_ production and iron accumulation in a mitochondrial metabolism-dependent manner^34^.

Additionally, it was shown that overexpression of ACO2 prevents H_2_O_2_-induced mtDNA damage and the resulting mitochondrial translocation of p53 and apoptosis in alveolar epithelial cells, while knockdown of ACO2 enhanced H_2_O_2_-induced mtDNA damage and subsequent apoptosis^35^. In the light of these results, we can conclude that the observed increased susceptibility of ACO2-mutant fibroblasts to H_2_O_2_-induced oxidative stress is linked to impaired ACO2 function and subsequent mitochondria-induced cell death.

Given the insufficiency of the ACO2 mutant protein to rescue the growth defect of *Δaco1*-yeast, together with the phenotypes described in ACO2-mutant fibroblasts and the autosomal dominant inheritance of the heterozygous mutation, we reason that the c.1699-1749del51 deletion causes a haploinsufficiency.

It is worth noting that temporal pallor of the optic nerve is a common trait of mitochondrial defects and autosomal dominant optic atrophy (ADOA; i.e. Kjer´s syndrome)^36,37^. Up to 70% of ADOA cases are caused by mutations in the *OPA1* gene^38^. Interestingly, recent studies showed that OPA1 and ACO2 are involved in similar mechanisms, thereby possibly explaining shared clinical phenotypes in patients carrying mutations in *ACO2* or *OPA1*. In particular, OPA1 regulates mtDNA integrity by tethering nucleoids to the inner mitochondrial membrane, subsequently influencing mtDNA replication and distribution^39,40^. Consequently, knockout of *OPA1* caused disorganization of the cristae and a decrease of mtDNA copy number in mouse embryonic fibroblasts^39^. Furthermore, knockdown of *OPA1* induced by siRNA in murine neurons led to a decrease of baseline mitochondrial respiratory function and aconitase enzyme activity while at the same time aconitase protein levels were not affected. In addition, neurons with *OPA1* knockdown were more susceptible to oxidative stress, resulting in elevated rotenone-induced cell death, likely due to an impairment of the ROS defence in these cells^41^. However, mutations in *OPA1* were excluded in the herein described patient.

In summary, our study provides evidence for a novel mutation in the *ACO2* gene causing haploinsufficiency in a pedigree with autosomal-dominant inherited optic nerve atrophy. Thereby, our study adds further details to the complex picture of mitochondrial defects as underlying cause of optic nerve atrophy caused by mutations in *ACO2*.

## Material and methods

### Clinical investigations, blood collection, informed consent

Informed consent was obtained from all participants. Venous blood samples were used to extract genomic DNA using standard protocols. The study was conducted in accordance with the principles of the Declaration of Helsinki and approved by the institutional review board of the Ethics Committee of the University Hospital of Tübingen, Germany (ref. 112/2001).

The index patient was examined at the Centre for Ophthalmology, University of Tübingen, Germany. The last follow-up examination was performed in 2014, including bst corrected visual acuity (BCVA), slit lamp examination and funduscopy in mydriasis, spectral domain optical coherence tomography (SD-OCT), static 30° perimetry (Octopus 900; Haag-Streit International, Wedel, Germany), full-field and multifocal electroretinography according to ISCEV (International Society for Clinical Electrophysiology of Vision) standards with an Espion E2/E3 (Diagnosys LLC, Cambridge, UK).

### Exome sequencing

We performed whole exome sequencing (WES) in a cohort of 9 unrelated patients with dominant inherited optic atrophy. Exomes were enriched using the SureSelect XT Human All Exon 50 Mb kit, versions 4 or 5 (Agilent Technologies, Santa Clara, CA, USA). Sequencing was performed on HiSeq 2500 systems (Illumina, San Diego, CA, USA). We considered a sub panel of genes, which are associated with optic atrophy: OPA1, OPA3, EM126A, WFS1, MFN2, SPG7, ACO2, RTN4IP1 and AFG3L2. Reads were aligned against the human assembly hg19 (GRCh37) using Burrows-Wheeler Aligner version 0.7.5^42^. We performed variant calling using SAMtools version 0.1.18^43^, PINDEL version 0.2.4t^44^ and ExomeDepth version 1.0.0^45^. Subsequently, variants were filtered using the SAMtools varFilter script and custom scripts. Shortly, only SNVs and indels in coding regions (nonsense, missense and canonical splice site variants as well as frameshift indels) having a potential effect on protein function in silico (assessed using predictions from PolyPhen-2 (https://genetics.bwh.harward.edu/pph2/), SIFT (https://sift.bii.a-star.edu.sg/) and CADD (https://cadd.gs.washington.edu/) were considered. From those, only private variants or those with a minor allele frequency < 1% in a cohort of more than 66,000 control individuals (ExAC Browser; https://exac.broadinstitute.org/); and 6742 in-house exomes were kept for subsequent analyses.

### Sanger sequencing

A 700 bp fragment encompassing exon 13 and 14 of the human ACO2 gene was amplified from genomic DNA with primers ACO2_Ex13F (5′ TTGGTAGGTGCAGGAGACAG 3′) and ACO2_Ex14R2 (5′ AAACCTCCCTTCCATCTCCC 3′) in a 25 µl PCR reaction with AmpliTaqII buffer (Applied Biosystems, Weiterstadt, Germany), 200 µM each dNTP, 200 nM each primer, 1 U Firepol Taq Polymerase (ATG Biosynthetics GmbH, Merzhausen, Germany), and 100 ng of total genomic DNA. PCR cycling was carried out with an initial denaturation for 4 min at 94 °C, 35 cycles of 20 s 95 °C, 30 s 59 °C and 90 s at 72 °C, and a final extension for 7 min at 72 °C. The PCR fragment was purified by ExoSAP treatment (Thermo Fisher Scientific GmbH, Dreieich, Germany) and used for cycle sequencing using BigDye 1.1 chemistry (Applied Biosystems) and nested primer ACO2_Ex14R1 (5′ GTTCATGGCCCTTCCCGAT 3′). Sequencing products were purified by isopropanol precipitation and separated on an ABI 3130XL sequencer (Applied Biosystems). Raw data were processed with SeqA 5.1 and sequence alignment was done using the SeqMan software (Lasergene, Madison, WI, USA).

### Quantitative cDNA analyses

Total blood RNA was reverse transcribed into single-stranded cDNA with the SuperScript II First-Strand Synthesis Kit (Invitrogen GmbH, Karlsruhe, Germany) using ACO2 gene-specific primers (oligonucleotide sequences available upon request).

### Structural analysis of deleted segment 571–583 in human ACO2

To predict the effects of deleting positions 571–583 in ACO2 we used a three-dimensional structure of the analyzed protein and inspected non-covalent interactions (hydrogen bonds, salt bridges) in the structure. The structure of human ACO2 (NP_001089.1) has not been solved yet. However, its amino acid sequence is 96.5% identical to the sequence of the same enzyme from pig (pdb entry 1b0j). Therefore, we used this structure as a template to predict the structure of human ACO2^14^. The structure was predicted using the homology-modelling software MODELLER^46^. The MolProbity web-server^47^ was then used to optimize side chain orientations and to add hydrogen atoms to the structure.

### Yeast drop dilution assay

The growth assay of yeast was done as described before^9,32^. Strains harbouring the appropriate plasmids were grown at 30 °C in synthetic depleted (SD) medium containing 0.67% (wt/vol) yeast nitrogen base (Difco, Detroit, MI) and 2% galactose or 3% ethanol supplemented with the appropriate amino acids (50 mg/ml) overnight, followed by drop dilution growth on the indicated media agar plates.

### Fibroblast cell culture

Informed consent was obtained from all individuals included in this study prior to skin biopsy collection. Skin biopsies were obtained from one male patient with the c.1699_1749del51 deletion in *ACO2.* Age- and gender-matched healthy control individuals were recruited from the Luxembourg Parkinson’s study and fibroblasts were provided by the Integrated Biobank Luxembourg (IBBL) within the framework of the National Centre for Excellence in Research on Parkinson’s disease (NCER-PD^48^). All fibroblasts were grown in cell culture approved flasks and plates (BD Bioscience, Heidelberg, Germany; Corning, Kaiserslautern, Germany; Greiner Bio-One GmbH, Frickenhausen, Germany; Thermo Fisher Scientific, Braunschweig, Germany) with DMEM +/+ medium (4.5 g/l Glucose + 15% FBS + 1% Pen/Strep) and were incubated at 37 °C and 5% CO_2_. Fibroblasts were splitted with trypsin–EDTA (0.25%) phenol red (Thermo Fisher Scientific, Braunschweig, Germany). Cell cultures were tested for mycoplasma contamination once per month using the Plasmo Test™ Detection Kit (InvivoGen). For all experiments, fibroblasts were used below passage 9.

### Western blot (WB) analysis

Fibroblasts were grown under standard conditions and lysed with RIPA buffer containing Complete Protease Inhibitor (Roche, Germany). For each sample, we harvested ~ 500,000 cells and loaded 30 µg of total protein on 10% polyacrylamide gels and resolved by one-dimensional discontinuous sodium dodecyl sulfate polyacrylamide gel electrophoresis (SDS-PAGE). Proteins were blotted on nitrocellulose membrane (Invitrogen GmbH, Karlsruhe, Germany) by using the iBlot 2 device (Invitrogen GmbH, Karlsruhe, Germany) for 7 min at 20 V. Proteins of interest were labeled with primary antibodies against ACO2 (anti-rabbit; Abcam: ab129069; dilution: 1:1000), β-Actin (anti-mouse; Cell signal: 37005; dilution: 1:5000), TOM20 (anti-rabbit; Santa Cruz: Sc-11415; dilution: 1:1000) and secondary antibodies goat anti-mouse IgG (Novex: A24524; dilution: 1:10,000) or goat anti-rabbit IgG (Novex: A24537; 1:5000), respectively. Protein bands were visualized with Amersham ECL Western Blotting Detection Reagent (GE healthcare, Freiburg, Germany) on the ODYSSEY chemiluminescence 2800 Fc (Li-COR, Lincoln, USA). Three independent samples per cell line (n = 3) were assessed. Image J software was used for relative signal quantification.

### Mitochondrial fractionation for measurement of Aconitase 2 activity

For measurement of ACO2 enzyme activity, mitochondrial fractions were prepared from fibroblasts as described before^49^. Fibroblasts were collected using trypsin–EDTA (0.25%) phenol red (Thermo Fisher Scientific, Braunschweig, Germany). Obtained cell pellets contained ~ 6 × 10^6^ to 10 × 10^6^ cells and were mixed with 240 µl homogenization buffer (10 mM Tris, pH7.4; 1 mM EDTA; 250 mM Sucrose; Complete Protease Inhibitor (Roche, Germany)) on ice. Cells were homogenized for 1 min and samples were subsequently mixed with homogenization buffer to a total volume of 4 ml. The homogenate was centrifuged for 10 min at 4 °C, 1500×*g*. The supernatant was centrifuged again for 10 min at 4 °C, 1500×*g*. The supernatant from this step was afterwards centrifuged for 10 min at 4 °C, 10,000 rpm and this step was repeated once. The resulting supernatant contained the cytoplasmic fraction and the pellet contained the crude mitochondria fraction.

### Biochemical measurement of Aconitase 2 activity

The Aconitase-340 assay (Bioxytech, Foster City, USA) was used to measure enzymatic activity of ACO2, according to the manufacturer’s protocol. Measurements were performed on the monochromatic spectrophotometer SPECORD 210Plus (Analytic Jena AG, Jena, Germany). Crude mitochondria fractions were used as described above. The OD values from the beginning (OD1) and the end (OD2) of the phase of linear increase was used to calculate the ACO2 enzyme activity. Absorption rate: $\Delta {A}_{340}/\text{min}= \frac{OD2-OD1}{time}$. Net rate was determined by subtraction of the blank rate form the samples rate: $Net \Delta {A}_{340}/\text{min}= \Delta {A}_{340}/\text{min} \left(Sample\right)-\Delta {A}_{340}/\text{min }(Blank)$. Aconitase activity (mU) was calculated in consideration of the molar extinction coefficient,$\varepsilon$, for NADPH ($\varepsilon$ (NADPH) = 6220 M^−1^ cm^−1^, the temperature correction coefficient, c (c = 2.4435) and the assay dilution, d (d = 4): Aconitase activity ($mU)= \frac{Net {A}_{340}/\text{min}}{c\cdot \varepsilon }\cdot d$. The enzyme activity of aconitase was corrected to the dilution and normalized to protein concentration of the sample. Sample Dilution Correction (mU_1_) = $\frac{mU}{Dilution }.$ Protein Correction: mU_2_ = $\frac{{mU}_{1}}{Protein \,concentration}.$

### mtDNA analysis

Mitochondrial major arc deletions, transcription-associated 7S DNA and copy number were quantified using a real-time PCR (RT-PCR) approach with TaqMan probes as previously described^50^. In brief, three probes targeting different regions within the mitochondrial genome are quantified simultaneously. A probe in the mtDNA gene *ND1*, which is located in the minor arc and typically spared from deletions, is measured relatively to a probe in the mtDNA gene *ND4*, which is located within the major arc in a region that is commonly affected by the 4977 bp deletion. In addition, with a probe targeting the mitochondrial D-loop, the abundance of 7S DNA is measured relative to *ND1* to determine the proportion of mtDNA molecules currently undergoing transcription^51^. Finally, the nuclear encoded single-copy gene *B2M* was used as a reference to quantify the amount of wildtype mtDNA copies (*ND1:B2M*).

### Seahorse–XF^e^96 extracellular flux analyser

#### Measurement of oxygen consumption rate (OCR)

We used whole cells to analyse OCR with the XF^e^96 extracellular flux Analyzer (Seahorse Bioscience, USA) as previously reported^52,53^. Twenty-four hours before the measurement, 14,000 cells per well were seeded into SF96 cell culture microplates (Seahorse Bioscience, USA) and incubated over night at 37 °C. The preparation of the assay cartridge, assay media and cell culture microplate for the measurement was done according to manufacturer’s protocol (Seahorse Bioscience, USA). During measurement, cells were subsequently treated with 1 µM Oligomycin, 250 nM FCCP and a 5 µM mixture of Rotenone and Antimycin A (all chemicals obtained from Sigma Aldrich, Germany). For normalization of OCR to total protein concentration per well, the cells were lysed in the well after OCR measurement, using a lysis buffer with Triton X-100 and Complete Protease Inhibitor (Roche Applied Science, Mannheim, Germany) for 10 min at room temperature. Protein concentrations were measured using Bradford solution (Bio-Rad Laboratories, Munich, Germany) according to the manufacturer’s protocol. OCR raw data were normalized to total protein concentration in each well.

#### Measurement of extracellular acidification rate (ECAR)

ECAR was measured in whole cells using the Glycolysis stress test on the XF^e^96 extracellular flux Analyzer according to the manufacturer’s protocol (Seahorse Bioscience, Santa Clara, USA). On the day before the measurement, 14,000 cells per well were seeded into SF96 cell culture microplates (Seahorse Bioscience, USA) and allowed to grow for 24 h. The assay media was composed of bicarbonate-free basal DMEM (Sigma Aldrich Chemie GmbH, Munich, Germany) and 1% l-Glutamine (Sigma Aldrich Chemie GmbH, Munich, Germany) without Glucose. During measurement, cells were sequentially treated with 1 mM Glucose (Sigma Aldrich Chemie GmbH, Munich, Germany), 10 µM Oligomycin (Sigma Aldrich Chemie GmbH, Munich, Germany) and 10 mM of the Glucose analogue 2-deoxyglucose (2-DG; Sigma Aldrich Chemie GmbH, Munich, Germany). ECAR raw data were normalized to total protein concentration per well, similar to OCR.

### Live cell imaging microscopy

For live cell imaging, fibroblasts were cultured under standard growth conditions and seeded into Nunc Lab-Tek Chamber slides (Thermo Fisher Scientific, Braunschweig, Germany). Fibroblasts were stained with 0.1 nM MitoTracker green FM (Thermo Fisher Scientific, Braunschweig, Germany) in DMEM+/+ for 20 min at 37 °C and 5% CO_2_. We used an Axiovert 2000 microscope with spinning disc (Carl Zeiss Microimaging GmbH, Jena, Germany), including an incubation chamber for the maintenance of a humidified atmosphere containing 5% CO_2_ at 37 °C during imaging. MatLab was used for data analysis on single cell level, using the parameters perimeter, area, major axis and minor axis in order to calculate the Form Factor (FF = (perimeter^2^)/(4*π*area)) as indicator of mitochondrial branching and Aspect Ratio (AR = major axis/minor axis) as indicator of mitochondrial length. For each cell line, between 75 and 85 cells were imaged and analyzed.

### Flow cytometry

Flow cytometry measurement was performed with the BD LSR Fortessa (Becton, Dickinson and Company, Ermbodegem, Belgium). At least 10,000 cells per sample were measured and analysed using the Flowjo software (Flowjo LLC, Oregon, USA). An unstained control was included in every experiment in order to determine the autofluorescence level of each fibroblast line. Fibroblasts were grown under standard conditions at a density of 200,000 cells per well in 6 well plates. For measurement of the mitochondrial membrane potential, cells were treated with 5 nM Valinomycin (Sigma Aldrich Chemie GmbH, Munich Germany) for 14 h. Fibroblasts were harvested with trypsin and stained with 20 µM TMRE (Thermo Fisher Scientific, Germany) for 20 min at 37 °C. For measurement of mitochondrial ROS, the growth medium was exchanged to low Glucose medium (DMEM 1.5 g/l Glucose + 1% Pen/Strep) without FBS 24 h prior to the experiment. Cells were subsequently treated with 20 nM Piericidin A (Santa Cruz, Dallas, Texas) for 14 h in order to inhibit complex I of the respiratory chain. Cells were harvested in trypsin and washed twice with HBSS (Thermo Fischer Scientific, Braunschweig, Germany). Then, cells were stained with 2.25 µM MitoSOX™ Red Mitochondrial Superoxide Indicator (Thermo Fischer Scientific, Braunschweig, Germany) for 20 min at 37 °C in a CO_2_-free incubator. Afterwards, cells were washed once with HBSS (Thermo Fischer Scientific, Braunschweig, Germany) before measurement.

### LDH cytotoxicity assay

The Lactate dehydrogenase (LDH) cytotoxicity assay (Thermo Fisher Scientific, Braunschweig, Germany) was used according to the manufacturer’s protocol in order to analyse cell viability. Fibroblasts were grown under standard conditions in 96 well plates at a density of 15,000 cells per well. Cells were treated with 5 mM hydrogen peroxide (H_2_O_2_) for 4 h at 37 °C. Colorimetric measurements were performed on the Microplate Cytation 5 M Cell imaging Multi Mode Reader (Bio-Rad laboratories GmbH, Munich, Germany).

### Statistical analyses

Graph-Pad Prism 8.0 software was used to assess the statistical significance. All measurements were independently repeated three times or more, as indicated in the figure legends (n indicates the number of independent biological replicates). In order to account for small sample size, we used non-parametric tests throughout as detailed in the figure legends.

## Supplementary information

Supplementary Information.

## Data Availability

The datasets generated and analyzed during the current study are available from the corresponding author on reasonable request.
