# Supplementary material for: Haploinsufficiency due to a novel ACO2 deletion causes mitochondrial dysfunction in fibroblasts from a patient with dominant optic nerve atrophy
Source: Sci Rep. 2020 Oct 7;10:16736. doi: 10.1038/s41598-020-73557-4 (PMC7541502; doi:10.1038/s41598-020-73557-4)
Supplement: Supplementary file 1 — Supplementary Information. [file 41598_2020_73557_MOESM1_ESM.pdf]

Haploinsufficiency due to a novel *ACO2* deletion causes mitochondrial dysfunction in fibroblasts from a patient with dominant optic nerve atrophy

Marie Anne-Catherine Neumann, Dajana Grossmann, Simone Schimpf-Linzenbold, Dana Dayan, Katarina Stingl, Reut Ben-Menachem, Ophry Pines, François Massart, Sylvie Delcambre, Jenny Ghelfi, Jill Bohler, Tim Strom, Amit Kessel, Abdussalam Azem, Ludger Schöls, Anne Grünewald, Bernd Wissinger, Rejko Krüger

Supplementary Figure 1:

```
Identity:     514/784 (65.6%)
Similarity:   618/784 (78.8%)

NP_001089.1     1 MAPYSLLVTR--LQKALGVRQYHVASVLCQRAKVAMSHFEPNEYIHYDLL     48
                       :|..|  :::.: ||.....|.|.:.:||..:..|.:.:|:|...
NP_013407.1     1 -----MLSARSAIKRPI-VRGLATVSNLTRDSKVNQNLLEDHSFINYKQN     44

NP_001089.1    49 EKNINIVRKRLNRPLTLSEKIVYGHLDDPASQEIERGKSYLRLRPDRVAM     98
                  .:.::|||||||||.|.:|||:|||||||..|:|:||.|||:|||||||.
NP_013407.1    45 VETLDIVRKRLNRPFTYAEKILYGHLDDPHGQDIQRGVSYLKLRPDRVAC     94

NP_001089.1    99 QDATAQMAMLQFISSGLSKVAVPSTIHCDHLIEAQVGGEKDLRRAKDINQ    148
                  ||||||||:|||:|:||.:||.|.|:||||||:|||||||||:||.|:|:
NP_013407.1    95 QDATAQMAILQFMSAGLPQVAKPVTVHCDHLIQAQVGGEKDLKRAIDLNK    144

NP_001089.1   149 EVYNFLATAGAKYGVGFWKPGSGIIHQIILENYAYPGVLLIGTDSHTPNG    198
                  |||:|||:|.|||.:|||||||||||||:|||||:||.|:|||||||||.
NP_013407.1   145 EVYDFLASATAKYNMGFWKPGSGIIHQIVLENYAFPGALIIGTDSHTPNA    194

NP_001089.1   199 GGLGGICIGVGGADAVDVMAGIPWELKCPKVIGVKLTGSLSGWSSPKDVI    248
                  ||||.:.||||||||||||||.|||||.||::||||||.::||:||||:|
NP_013407.1   195 GGLGQLAIGVGGADAVDVMAGRPWELKAPKILGVKLTGKMNGWTSPKDII    244

NP_001089.1   249 LKVAGILTVKGGTGAIVEYHGPGVDSISCTGMATICNNGAEIGATTSVFP    298
                  ||:|||.|||||||.||||.|.|||:.|.|||.|||||||||||||||||
NP_013407.1   245 LKLAGITTVKGGTGKIVEYFGDGVDTFSATGMGTICNMGAEIGATTSVFP    294

NP_001089.1   299 YNHRMKKYLSKTGREDIANLADEF-KDHLVPDPGCHYDQLIEINLSELKP    347
                  :|..|.:||..|||..||:.|..: ||.|..|....||:::||:|:.|:|
NP_013407.1   295 FNKSMIEYLEATGRGKIADFAKLYHKDLLSADKDAEYDEVVEIDLNTLEP    344

NP_001089.1   348 HINGPFTPDLAHPVAEVGKVAEKEGWPLDIRVGLIGSCTNSSYEDMGRSA    397
                  :||||||||||.||:::.:||....||||:||||||||||||||||.|||
NP_013407.1   345 YINGPFTPDLATPVSKMKEVAVANNWPLDVRVGLIGSCTNSSYEDMSRSA    394

NP_001089.1   398 AVAKQALAHGLKCKSQFTITPGSEQIRATIERDGYAQILRDLGGIVLANA    447
                  ::.|.|.|||||.|:.||:|||||||||||||||..:..::.||||||||
NP_013407.1   395 SIVKDAAAHGLKSKTIFTVTPGSEQIRATIERDGQLETFKEFGGIVLANA    444

NP_001089.1   448 CGPCIGQWDRKDIKKGEKNTIVTSYNRNFTGRNDANPETHAFVTSPEIVT    497
                  ||||||||||:|||||:|||||:|||||||.|||.||:|||||.|||:||
NP_013407.1   445 CGPCIGQWDRRDIKKGDKNTIVSSYNRNFTSRNDGNPQTHAFVASPELVT    494

NP_001089.1   498 ALAIAGTLKFNPETDYLTGTDGKKFRLEAPDADELPKGEFDPGQDTYQHP    547
                  |.||||.|:|||.||.|...||.:|.|:.|..|.||:..:|.|::|||.|
NP_013407.1   495 AFAIAGDLRFNPLTDKLKDKDGNEFMLKPPHGDGLPQRGYDAGENTYQAP    544

NP_001089.1   548 PKDSSGQHVDVSPTSQRLQLLEPFDKWDGKDLEDLQILIKVKGKCTTDHI    597
                  |.|.|...|.|||||.|||||:||..|||||.:|:.||||..||.|||||
NP_013407.1   545 PADRSTVEVKVSPTSDRLQLLKPFKPWDGKDAKDMPILIKAVGKTTTDHI    594

NP_001089.1   598 SAAGPWLKFRGHLDNISNNLLIGAINIENGKANSVRNAVTQEFGPVPDTA    647
                  |.||||||:||||:|||||.:|||||.||.|||.|:|..|.|:..|||||
NP_013407.1   595 SMAGPWLKYRGHLENISNNYMIGAINAENKKANCVKNVYTGEYKGVPDTA    644

NP_001089.1   648 RYYKKHGIRWVVIGDENYGEGSSREHAALEPRHLGGRAIITKSFARIHET    697
                  |.|:..||:||||||||:||||||||||||||.|||.|||||||||||||
NP_013407.1   645 RDYRDQGIKWVVIGDENFGEGSSREHAALEPRFLGGFAIITKSFARIHET    694

NP_001089.1   698 NLKKQGLLPLTFADPADYNKIHPVDKLTIQGLKDFTPGKPLKCIIKHPNG    747
                  ||||||||||.|.:||||:||:|.|::.|.||.:..||||:...:...||
NP_013407.1   695 NLKKQGLLPLNFKNPADYDKINPDDRIDILGLAELAPGKPVTMRVHPKNG    744

NP_001089.1   748 TQETILLNHTFNETQIEWFRAGSALNRMKELQQ-    780
                  .....:|.||||:.|||||:.|||||::|..::
NP_013407.1   745 KPWDAVLTHTFNDEQIEWFKYGSALNKIKADEKK    778
```

Multiple sequence alignment of human ACO2 (NP_001089.1) and yeast ACO1 (NP_013407.1) generated by MUSCLE version 3.6 using the option maxiters 2 [Edgar, R.C. MUSCLE: multiple sequence alignment with high accuracy and high throughput. Nucleic Acids Res. 32, 1792-7 (2004)]. Residues interacting with the substrate are highlighted in green [Lauble, H. et al., Crystal structures of aconitase with isocitrate and nitrocitrate bound. Biochemistry 31, 2735-48 (1992)]. The deletion site is highlighted in red.

Supplementary Figure 2:

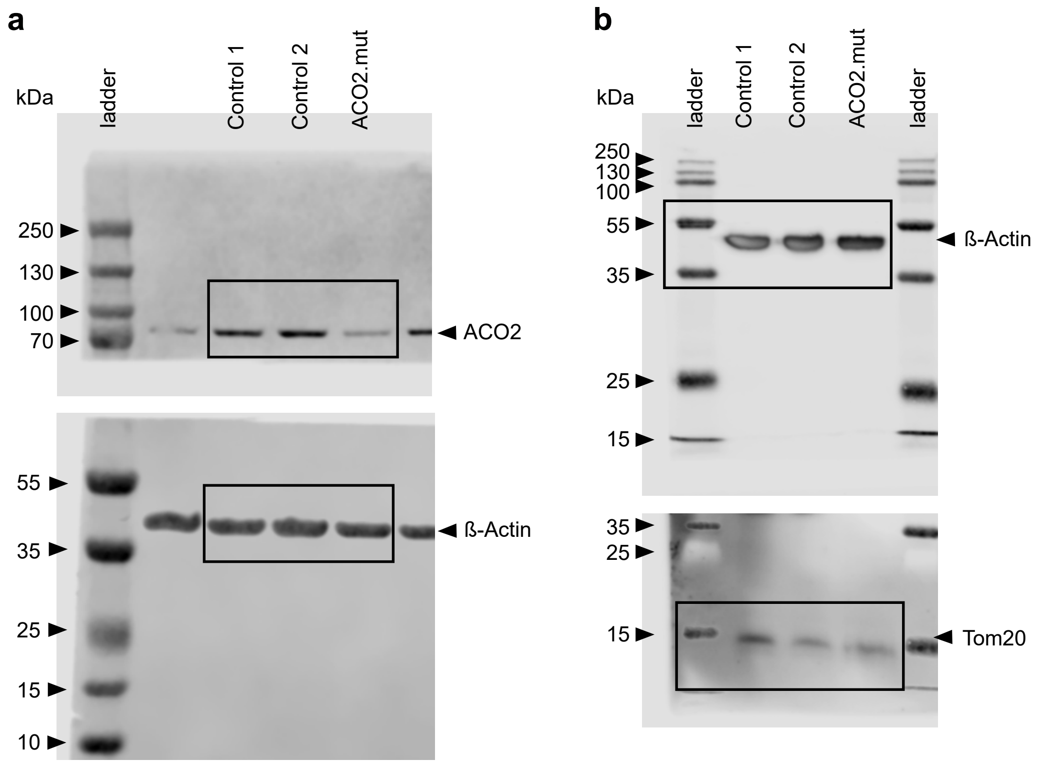

a) Image of Western blot membranes for fibroblast samples Control 1, Control 2 and ACO2.mut probed with antibodies against ACO2 and β-Actin. Black boxes indicate the image sections shown in Figure 3A. b) Image of Western blot membranes of fibroblast samples Control 1, Control 2 and ACO2.mut were probed with antibodies against β-Actin and Tom20. Black boxes indicate the image sections shown in Figure 3C. Bands of the protein ladder are indicated on the right or the left side of the images. The size of the protein ladder bands are indicated in kDa and black arrow heads. The identity of the samples is indicated at the top of each membrane.
